# Advanced Polymer Simulation and Processing

**DOI:** 10.3390/polym14122480

**Published:** 2022-06-18

**Authors:** Célio Fernandes, Salah A. Faroughi, Luís L. Ferrás, Alexandre M. Afonso

**Affiliations:** 1 LASI-Associate Laboratory of Intelligent Systems, Institute for Polymers and Composites, Polymer Engineering Department, School of Engineering of the University of Minho, Campus of Azurém, 4800-058 Guimarães, Portugal; 2 Geo-Intelligence Laboratory, Ingram School of Engineering, Texas State University, San Marcos, TX 78666, USA; salah.faroughi@txstate.edu; 3 Center of Mathematics (CMAT), University of Minho, Campus de Azurém, 4800-058 Guimarães, Portugal; lferras@fe.up.pt; 4 CEFT, Department of Mechanical Engineering, University of Porto, 4200-465 Porto, Portugal; aafonso@fe.up.pt

Polymer processing techniques are of paramount importance in the manufacture of polymer parts. The key focus is on producing parts with the desired quality, which usually refers to mechanical performance, dimensional conformity, and appearance. To maximize the overall efficiency of polymer processing techniques, advanced modeling codes are needed along with experimental setups to simulate and optimize the processes.

To improve modeling codes for polymer processing techniques, Fernandes et al. [1] developed an incompressible, non-isothermal finite volume method based on the arbitrary Lagrangian–Eulerian formulation (ALE) to calculate the viscous flow of polymer melts obeying the Herschel–Bulkley constitutive equation. The new method is employed to compute the extrudate swell ratio for Bingham and Herschel–Bulkley flows (shear thinning and shear thickening). Spanjaards et al. [2] numerically investigated the effect of thixotropy on the swelling of a 2D planar extrudate for constant and fluctuating flow rates, and the effect of thixotropy on the swelling behavior of a 3D rectangular extrudate for a constant flow rate. It was concluded that the presence of a low-viscosity outer layer and a high-viscosity core in the die has a pronounced effect on the swelling ratio for thixotropic fluids. Marschik et al. [3] proposed new leakage–flow models that allow the effect of flight clearance to be included in the analysis of the melt-conveying zones of the extrusion process. They derived regression models to locally predict the shear-thinning flow through the flight clearance. Castelo et al. [4] proposed a moving least squares meshless interpolation technique to simulate Newtonian, generalized Newtonian, and viscoelastic fluid flows. The code verification and testing were performed using numerical stabilizers for the Oldroyd-B flow solution in a 2D cavity and for a Phan–Thien–Tanner fluid in a complex 3D geometry. Faroughi et al. [5] developed a meta-model using a stacking technique to accelerate the calculation of the drag coefficient of a spherical particle moving through viscoelastic fluids. The meta-model combines random forest (RF), extreme gradient boosting (XGBoost), and deep neural network (DNN) models and outputs a prediction based on the individual learner’s predictions and a DNN meta-regulator. The meta-model consistently outperformed the individual models in predicting the drag ground truth, and it provided accurate prediction in just a fraction of time compared with the conventional drag calculation. Huang et al. [6] proposed an artificial backpropagation neural network (BPNN) to render result predictions for the injection molding process. By inputting the plastic temperature, mold temperature, injection speed, holding pressure, and holding time in the molding parameters, the end of filling pressure, maximum cooling time, warpage, and shrinkage were accurately predicted by the BPNN. Baral et al. [7] proposed an accurate ab initio molecular dynamics and density functional theory calculations to investigate the structure and properties of the arginine–glycine–aspartate (RGD) sequence. The microscopic parameters determined from the quantum mechanical calculations proved useful in defining the range and strength of the complex molecular interactions between the RGD peptide and the integrin receptor. The study is also important in the context of conditions prevailing in the human body and relevant to health issues.

With a view to improving experimental setups for polymer processing techniques, Drozdov et al. [8] developed constitutive equations for the thermo-mechanical behavior of poly(ether ether ketone) under uniaxial deformation based on experimental observations made for uniaxial tensile tests, relaxation tests, and creep tests at various stresses in a wide temperature range. The activation energies for the elastoplastic, viscoelastic, and viscoelastoplastic responses adopt similar values at temperatures above the glass transition point. Castéran et al. [9] used the extrusion process for the controlled thermo-mechanical degradation of polyethylene in recycling applications. A Carreau–Yasuda model was developed to predict the rheological behavior based on in-line measurements of the die pressure of a reactive extrusion process. The linear viscoelastic behaviors were also used to predict the molecular weight distributions of the final products by an inverse rheological method. In addition, support vector machine regression (SVR) and sparse proper generalized decomposition (sPGD) techniques were chosen to predict the process outputs. Hirsch et al. [10] experimentally and numerically investigated the anisotropic mechanical behavior of a hybrid injection molding process using a continuous fiber-reinforced thermoplastic. The prediction of the mechanical behavior of the hybrid test structure under flexural loading by numerical simulation was significantly improved, resulting in a reduction in the deviation between the numerically predicted and experimentally measured flexural strength. Torres-Alba et al. [11] developed a hybrid cooling model based on the use of newly designed fluted conformal cooling channels combined with inserts of Fastcool material. The obtained results are in line with the sustainability criteria for green molds, which focus on reducing the cycle time and improving the quality of the complex molded parts. Li et al. [12] proposed a method to predict the warpage of crystalline parts molded by the rapid heat cycle molding process. Multi-layer models were created to predict the warpage with the same thicknesses as the skin–core structures in the molded parts. The numerical prediction results were compared with the experimental results, which showed that the average errors between the predicted warpage and the average experimental warpage were less than 10%. Lozano et al. [13] reviewed additive manufacturing (AM) technology to produce customized products with more complex geometries and short life cycles (flexibility) to keep up with the new variables imposed by the manufacturing environment. They address specific issues related to the characterization of the injection molding materials and molds most commonly used in this type of technology, their mechanical properties (part and mold), designs for all types of geometries, and costs. In addition, they highlight the advantages of this alternative manufacturing process, which is considered a desirable technology worldwide.

The editors are confident that this book will help researchers further understand the principles of polymer processing techniques, such as extrusion and injection molding, from both numerical and experimental perspectives. This book is also a useful resource for readers interested in the latest technologies, such as additive manufacturing. It will further advance the development of and improvements in data-driven algorithm research and promote the overlap and integration with polymer processing techniques.
